# Closed-Loop Spinal Cord Stimulation in Chronic Pain Management: Mechanisms, Clinical Evidence, and Emerging Perspectives

**DOI:** 10.3390/biomedicines13051091

**Published:** 2025-04-30

**Authors:** Nicholas Mangano, Andrew Torpey, Catherine Devitt, George A. Wen, Christopher Doh, Abhishek Gupta

**Affiliations:** 1 Department of Anesthesiology, Stony Brook Medicine, 101 Nicolls Road, Stony Brook, New York, NY 11794, USA; 2 Renaissance School of Medicine, Stony Brook University, 101 Nicolls Road, Stony Brook, New York, NY 11794, USA; 3 Department of Anesthesiology, Division of Chronic Pain, Stony Brook Medicine, 101 Nicolls Road, Stony Brook, New York, NY 11794, USA

**Keywords:** closed-loop spinal cord stimulation, spinal cord stimulation, chronic pain, lower back pain, neuromodulation, evoked compound action potentials, pain management, evoke, avalon, cost-effectiveness

## Abstract

**Background:** Chronic pain remains a major clinical challenge, which is often resistant to conventional treatments. Spinal cord stimulation has been used for decades to manage refractory pain, traditionally relying on open-loop systems with fixed-output stimulation. However, these systems fail to account for physiological variability, leading to inconsistent pain relief. Closed-loop spinal cord stimulation represents a significant advancement by utilizing evoked compound action potentials to continuously modulate stimulation intensity in real-time, ensuring more stable and effective pain management. **Methods**: A comprehensive literature review was conducted using PubMed and ClinicalTrials.gov to identify and synthesize relevant published and ongoing studies with a focus on open-loop spinal cord stimulation for managing lower back pain. **Results**: Clinical trials, including the Avalon and Evoke studies, have demonstrated that closed-loop spinal cord stimulation provides superior pain relief, functional improvement, and reduced opioid dependence compared to traditional open-loop systems. Patients receiving closed-loop stimulation reported significantly higher rates of sustained pain reduction, improved quality of life, and fewer complications related to overstimulation. Emerging studies suggest its potential for conditions beyond back pain, such as neuropathic pain, cancer-related pain, and Raynaud’s phenomenon. Furthermore, cost-effectiveness analyses indicate that closed-loop spinal cord stimulation is a more economically viable treatment option compared to conventional medical management and open-loop systems. **Conclusions**: Closed-loop spinal cord stimulation represents a transformative development in neuromodulation, offering personalized and adaptive pain management that is distinct from open-loop spinal cord stimulation. Further research is warranted to explore its long-term durability, broader applications, and integration with emerging technologies in pain management.

## 1. Introduction

Spinal cord stimulation (SCS) has been a treatment for chronic neck and low back pain for decades. It is well known that the target of SCS therapy has been the dorsal columns of the spinal cord. The current theory regarding the mechanism of action of SCS treatment continues to be multifaceted [1]. Based on the widely cited “gate control theory”, SCS involves stimulation of the substantia gelatinosa, which then modulates sensory input to the brain via large-diameter Aβ fibers. This stimulation, then, effectively “closes the gate”, inhibiting noxious stimuli [2]. SCS therapy has also been shown to alter the spinal cord at the neurochemical level. More specifically, SCS therapy stimulates the release of γ-aminobutyric acid, an inhibitory neurotransmitter, and a reduction in glutamate, an excitatory neurotransmitter, both of which collectively reduce pain transmission [3].

Traditional SCS systems deliver a stimulus to the spinal cord with a fixed output. These systems do not account for large variations in anatomy and movement or the physiological activity of the spinal cord. Closed-loop SCS (CL-SCS) is a novel advancement in SCS technology that delivers a more focused and continually adapting electrical stimulation. As the stimulus induces a suprathreshold response, the closed-loop system measures evoked compound action potentials (ECAPs). ECAPs are a result of therapeutic spinal cord activation. This pulse creates a feedback loop that autonomously and consistently adjusts the ECAP amplitude [4]. Therefore, CL-SCS may provide a greater reduction in chronic pain and clinical outcomes, not only by improving activities of daily living and function but also by reducing SCS trial times and user/product interference.

## 2. Methodology

A comprehensive literature review was conducted to identify relevant studies pertaining to lower back pain and spinal cord stimulation. Searches were performed using PubMed and ClinicalTrials.gov to capture both published peer-reviewed articles and registered clinical trials. The search strategy included combinations of Medical Subject Headings (MeSH) and relevant keywords related to open and closed-loop spinal cord stimulation, chronic lower back pain, evoked compound action potentials, and specific clinical trials evaluating closed-loop spinal cord stimulation. The inclusion criteria were limited to studies published in English, involving human subjects (or animals for experimental spinal cord stimulation techniques), and conducted primarily within the past 10 years. ClinicalTrials.gov (accessed on 1 March 2025) was additionally used to identify ongoing or unpublished studies to ensure the review captured the most current evidence. Titles and abstracts were screened independently by the authors, and full texts were assessed for relevance based on predefined eligibility criteria. The data were then analyzed and synthesized to provide a narrative summary of the findings.

## 3. Current State of Lower Back Pain

### 3.1. Epidemiology

Lower back pain is one of the most common symptoms experienced by patients of all demographics worldwide [5]. It is the fifth most common reason for seeking medical care [6]. According to the Global Burden of Diseases, Injuries, and Risk Factors Study, a comprehensive assessment of health loss across 195 countries since 1990 found that low back pain is one of the leading causes of years lived with disability in all ages [5]. The prevalence of low back pain has increased by 30% since the study began in 1990, representing more than 42 million years lived with disability cumulatively [5]. Though low back pain affects all ages and both sexes, its prevalence is highest in females aged 40 to 80 years old [7]. For the adult general population, the point prevalence of back pain is 11.9%, the one-month prevalence is 23.3%, and the lifetime prevalence is as high as 65–80% [6,7]. Interestingly, low back pain is more common in high-income countries [7]. In terms of occupational differences, low back pain is more common in patients who have physically demanding jobs [8]. Risk factors for developing low back pain include cardiovascular hypertension, smoking, overweight, and obesity [8]. Psychosocial factors also contribute to the chronicity of low back pain [8]. Low back pain has economic implications at both an individual and national level. It is the leading cause of activity limitation and work absence in many countries [9]. Individuals with chronic low back pain accumulate less wealth overall than those without back pain [10]. Additionally, low back pain is one of the most common reasons for early retirement. More people leave the workplace due to back pain than due to heart disease, diabetes, hypertension, neoplasm, respiratory disease, and asthma combined [10]. On a national scale, annual expenditures for the management of patients with low back pain exceed USD 100 billion. Of this, two-thirds are due to indirect costs, including loss of productivity [11].

### 3.2. Pathophysiology

The pathophysiology of chronic pain consists of complex multifactorial processes. To appreciate the mechanisms and utility of various treatment options for chronic low back pain, it is essential to understand pain pathways. In brief, pain starts with the detection of a noxious stimulus by a sensory receptor, creating an action potential. This action potential is transduced by a sensory nerve to the dorsal root ganglion of the spinal cord. The action potential is then transmitted via the spinal cord to the brain, leading to conscious subjective perception of pain [12]. Pain can be modulated at each step in this process, which ultimately changes the subjective experience of pain. Within the spinal cord, pain can be modulated by either excitatory or inhibitory interneurons or by descending inhibitory tracts [12]. A wide variety of underlying pathologies can lead to the development of chronic low back pain. Low back pain can be classified as mechanical or radicular [11]. Radicular pain stems from pathology of the spinal nerve roots. The two most common conditions that are associated with radicular pain are herniated discs and spinal stenosis. Mechanical pain refers to pain related to the musculoskeletal structures of the back. Common causes of mechanical pain include muscle strain, osteoarthritis, and spondyloarthropathies [11]. Psychosocial factors are associated with an increased risk of the chronicization of back pain. These factors include depressive mood, anxiety, stress, low job satisfaction, fear-avoidant behavior, catastrophizing, overreliance on medication, expectation of passive treatment, substance use, helplessness, and hopelessness [8,13,14,15].

### 3.3. Pharmacological and Non-Pharmacological Treatment Strategies

Treatment options for chronic low back pain aim to interrupt or modulate the pain pathway at various steps. Pharmacologic treatment options include over-the-counter agents such as acetaminophen and non-steroidal anti-inflammatory drugs, which have been shown to be effective for short-term relief [6]. Prescription medications that are commonly used in the treatment of back pain include skeletal muscle relaxants, opioids, tricyclic antidepressants, and serotonin–norepinephrine reuptake inhibitors [6]. Since part of the pain pathway includes conscious perception in the somatosensory cortex, psychological treatments can also be used in a multimodal approach to chronic low back pain. Cognitive behavioral therapy, biofeedback, and progressive relaxation have been shown to improve pain and function in patients with chronic low back pain [6]. Physical therapy and rehabilitation are generally first-line treatment options and have been shown to be effective in improving pain, reducing disability, and improving mood [6]. Some patients may also benefit from interventional options for the treatment of pain. Examples include trigger point injections, medial branch nerve blocks, radiofrequency ablations, and epidural steroid injections [6].

If these treatment options fail, spinal cord stimulation therapy can be considered. Spinal cord stimulators have been studied in a wide array of conditions that cause chronic back pain. They have been shown to benefit patients with chronic back and leg pain [16] intractable to conservative therapy caused by persistent spinal pain syndrome (formerly failed back surgery syndrome) [17], diabetic neuropathy [18,19], refractory angina [20], and chronic neuropathy [19]. As noted by Miękisiak, persistent spinal pain syndrome, one of the most common reasons for spinal cord stimulator placement, refers to “lumbar pain of unknown origin which is triggered or exacerbated by spinal surgery in the same topographical location” [21]. Persistent spinal pain syndrome develops when a surgery or trauma causes local tissue damage and inflammation, setting off a cascade of cytokines, prostaglandins, and bradykinin. The inflammatory response causes nerve irritation, resulting in pain. This pain can persist even after the initial cause of the pain is removed. Occasionally, scar tissue develops, which further irritates the spinal nerves. This process results in abnormal pain signaling. Prolonged pain leads to central sensitization, meaning that the brain and spinal cord perceive pain at lower thresholds [21]. This cycle is key in the development of chronic pain. SCS therapy aims to break this cycle to improve symptoms in patients with chronic pain.

## 4. Principles of Closed-Loop Spinal Cord Stimulation

When Melzack and Wall demonstrated that large-diameter non-nociceptive Aβ fibers (responsible for touch and pressure sensation) depolarize terminal branches and inhibit the propagation of nociception carried by the smaller C and Aδ fibers, they laid the foundation for the development of SCS [3,22,23]. Soon, Shealy et al. provided experimental evidence in animal models that the electrophysiologic pain response could be inhibited by electrical stimulation of the dorsal columns or anterolateral spinal cord, hypothesizing that targeted electrical stimulation of these fibers could suppress pain perception in human patients suffering from chronic pain conditions [24]. These findings quickly translated to clinical practice when Shealy implanted the first electrodes near the dorsal columns of patients with chronic intractable pain, delivering promising pain relief [25,26]. This “tonic stimulation” is applied with a frequency between 40 to 80 Hz and with a pulse width of 20 to 500 μs [27]. Over the following decades, technological advancements such as percutaneous electrode placement and multichannel devices improved patient outcomes [28,29]. However, despite these innovations, early SCS systems remained open-loop, requiring manual adjustments by patients or clinicians to control stimulation intensity in response to movement between standing and lying positions.

By the 1990s, open-loop SCS (OL-SCS) systems demonstrated improved reliability and long-term pain relief, with studies reporting that over half of patients achieved sustained benefit [29]. Yet, significant challenges persisted. One of the most persistent issues with open-loop SCS was electrode migration, which disrupted the delivery of optimal stimulation, requiring surgical revision of electrode position [28]. Furthermore, lead fractures [29], electrode migration, wire breakage [30], electrode dislocation [31], and receiver failures [28] continued to complicate long-term device reliability. Even when electrodes remained properly anchored, postural changes caused unpredictable shifts in stimulation intensity. Magnetic resonance imaging (MRI) studies confirmed that at the lower thoracic level, the dorsomedial layer increases by 2.2 to 3.4 mm, and that the distance between spinal and vertebral midline may vary by 1.5 to 2 mm in either direction [32]. Clinically, as many as 95% of patients experienced significant differences in stimulation needs when moving between lying, sitting, and standing [33]. Even dynamics like heartbeat and breathing can alter stimulation intensity [4]. These fluctuations resulted in either understimulation, reducing therapeutic efficacy, or overstimulation, leading to discomfort. These challenges underscore the need for CL-SCS, a self-adjusting system to modulate stimulation parameters and maintain therapeutic efficacy.

The ECAP provided the breakthrough necessary for closed-loop feedback by measuring the voltage change in dorsal column fibers following an electrical pulse, serving as a real-time proxy for neural activation. As opposed to the discussed open-loop system, where the physician or patient must manually adjust stimulation dosage, the ECAP-controlled closed-loop system self-modulates, adjusts in real-time, and delivers therapeutic stimulation based on distance changes between the spinal cord and electrode. On an oscilloscope, the spinal ECAP typically displays a triphasic shape, comprising a small positive spike (P1), a sharp negative spike (N1), and a large positive spike (P2). The amplitude of the ECAP is defined as the voltage difference between P2 and N1 spikes and correlates quantitatively with the total number of action potentials induced that activate pain-inhibiting neurons. Interestingly, the P1 is not observed in all patients and can be observed as two peaks in some, while the P2 wave is sometimes separated by a negative dip. These waves can be induced or altered by the effect of the patient and the intensity of stimulation [34]. Modeling has also demonstrated that ECAPs are produced by dorsal column neurons of 8.7 to 10 μm diameter [35]. Spinal ECAPs can be measured with every stimulation pulse, processed by the implanted stimulator, and modulated by subsequent pulses. Rather than requiring manual adjustments, ECAP-controlled CL-SCS dynamically modulates stimulation intensity within a therapeutic window. This is particularly responsive as quantitative feedback for movements that result in abrupt changes in stimulation distance without postural change (e.g., cough, sneeze, breathing) [36].

One of the key mechanisms of tonic SCS involves the activation of inhibitory interneurons in the dorsal horn, which release the neurotransmitter gamma-aminobutyric acid (GABA). A key study in rats suggested that 30 min of SCS therapy resulted in increased release of GABA from GABA-immunoreactive interneurons, contributing to the suppression of pain transmission [37]. Beyond this, tonic SCS also exerts effects through supraspinal pathways, particularly through descending serotonergic modulation. Through “top-down” modulatory circuits, areas such as the rostral ventromedial medulla in the brainstem increase serotonin release onto the dorsal horn [38]. Early work has shown that serotonin release in the spinal cord activates serotonin receptors, leading to pain suppression [39]. In the context of SCS, immunohistochemical stains demonstrated that stimulation increased serotonin content in the dorsal horns of rats ipsilateral to sites of nerve injury [40], producing pain relief in responding rats.

Furthermore, emerging bioinformatics analyses reveal that pain and spinal cord stimulation influence a wide range of biochemical pathways and cellular processes within the spinal cord, demonstrating complex neuromodulatory mechanisms. Importantly, it has been demonstrated that glial cells outnumber neurons 20 to 1 between T8 and T11 and assist in maintaining homeostasis between neurons. Chronic pain may thus intimately involve inflammatory mediators released by reactive microglia [41], while it has been demonstrated that glial cells can be electrically depolarized by an external electrical force [42,43]. Using differential stimulation in animals, the authors of a recent transcriptomic study demonstrated that spinal cord stimulation drives microglial gene expression toward a “naive” state, while exerting subtler effects on astrocytes, oligodendrocytes, and neurons [44]. Proteomic analyses further support this, showing decreased levels of pro-inflammatory proteins such as farnesyl transferase, alongside increased expression of anti-inflammatory markers, including mannose receptor 1 in microglia and ephrin B1 in astrocytes [45]. These findings suggest that SCS helps to recondition the neuro–glial interface to reduce inflammation and support recovery.

In addition to the aforementioned immunomodulatory effects, a recent proteomic study by Tilley and colleagues demonstrated that SCS most significantly enriched pathways involved in collagen fibril and extracellular matrix organization. This may play a role in maintaining the structural integrity and function of neural networks in the spinal cord. Simultaneously, SCS abrogated the expression of stress-induced mitogen-activated protein kinase 10, an inflammatory protein that has been implicated in chronic pain [46]. On the metabolic level, results suggest that SCS normalizes energy metabolism, with proteins involved in mitochondrial function and redox homeostasis, such as cytochrome c oxidase, with NADH dehydrogenase iron-sulfur protein being downregulated to physiologic levels after stimulation. These changes indicate a reduction in intracellular metabolic dysregulation, often seen in chronic pain states.

## 5. Periprocedural Considerations for SCS Therapy

### 5.1. Pre-Procedural Care

Like many other neuraxial procedures, SCS trials and implantations require adequate preoperative work-up. Patients should be asked about their medical history, including chronic pain symptoms, previous treatments, and other underlying conditions such as diabetes, cardiovascular disease, sleep apnea, and bleeding disorders. Laboratory testing may include a complete blood count, chemistry, the prothrombin time, and the partial thromboplastin time to assess for renal and hepatic disease, as well as coagulation status [47]. Compatibility with SCS should be established for patients with cardiac pacemakers and internal cardiac defibrillators prior to a spinal cord stimulator trial. Though newer pacemakers have little interaction with SCS devices, patients with defibrillators require closer observation [48]. Prior to SCS placement, psychological screening should be performed and may be required by insurance companies for approval [49]. Patients should also be assessed for underlying mood disorders and for discussion of their expectations of the procedure. High levels of depression, anxiety, dysfunctional coping, and somatization were associated with worse outcomes after lumbar surgery and SCS [50]. Though these diagnoses are not strict contraindications, psychological conditions should be treated prior to SCS therapy or other similarly invasive procedures.

Patients should be assessed for any contraindications to the insertion of a spinal cord stimulator, such as an active infection, severe spinal deformities, or conditions that may prevent appropriate electrode placement. Cervical, thoracic, and lumbar imaging (MRI or computed tomography) should be obtained to help plan for the placement of leads and should be reviewed to anticipate technical difficulty. Spine imaging studies are also helpful for ruling out contraindications like spinal tumors or structural abnormalities. Active systemic or local infection is a contraindication to spinal cord stimulator trials and implantations. Prior to neuromodulation trials and implants, remote infections should be identified and treated [51].

### 5.2. Perioperative Care

Informed consent should be obtained, and the patient’s ability to remain still, tolerate the prone position, and airway management should be considered, since these factors may affect the choice of anesthetic and surgical management [52]. SCS trials are often performed in the prone position with local anesthesia and sedation, as needed. Percutaneous SCS under general anesthesia (GA) with neuromonitoring can also be considered. Many providers prefer sedation over GA to help prevent nerve damage by allowing patients to report pain intraoperatively during needle insertion, lead placement, and testing for paresthesia [53]. Once paresthesia testing has been completed, the level of sedation may be increased. Deeper sedation may be required to avoid discomfort while creating the tunneling track for the implantable pulse generator (IPG).

**Figure 1:**
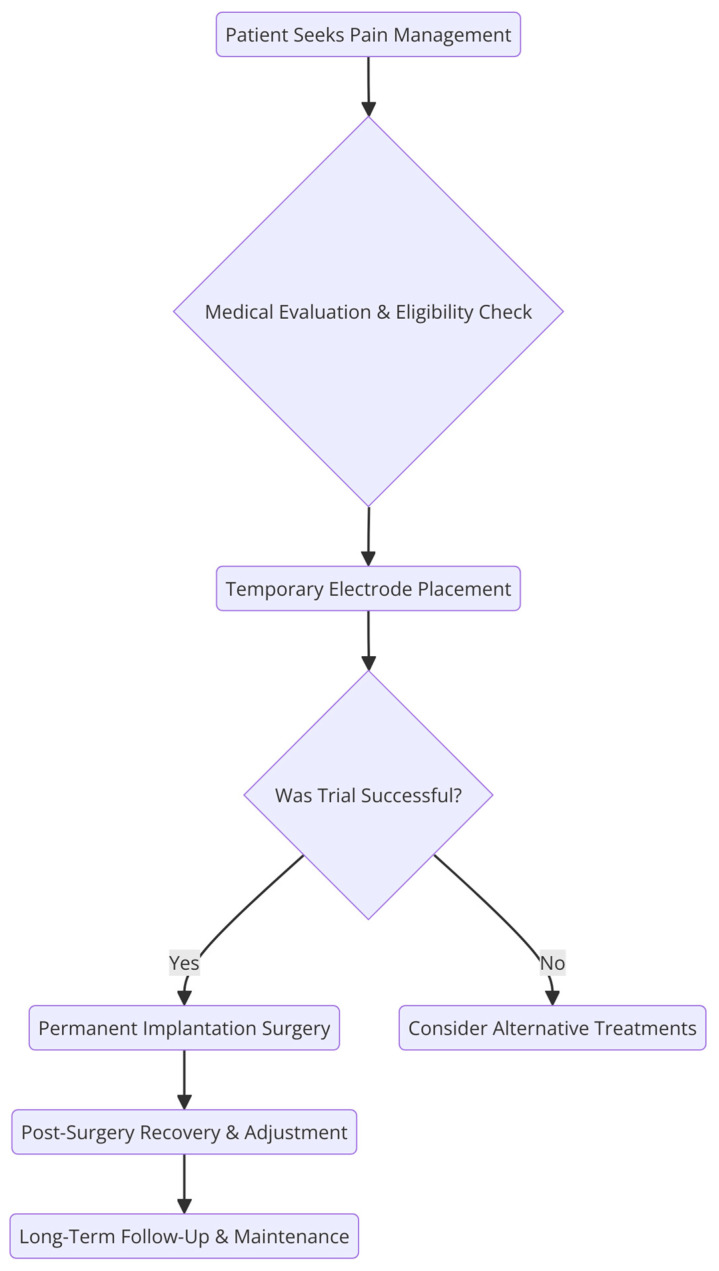
Traditional SCS treatment strategy.

Intraoperatively, the proceduralist should maintain a sterile technique with full surgical skin preparation and draping, as well as preoperative administration of antibiotics following institutional guidelines to reduce infection risk [54]. SCS devices are placed in two stages under fluoroscopic guidance with a trial period to test efficacy and followed by implantation if patients undergo a successful trial (see Figure 1). Radiographic imaging can guide optimal lead placement in real time [48]. While there is no consensus on trial length, on average, it lasts three to seven days. Patients requiring anticoagulation or antiplatelet medications may require a shorter trial length to minimize the risk of thrombotic events [48]. After a successful SCS trial, the implantation process involves creating a small incision to insert the epidural needles. The leads are then advanced into the epidural space. Once they are at the appropriate spinal level, the leads are anchored and may be tested for paresthesia. For paresthesia-based lead placement, the paresthesia should overlap with at least 80% of the pain distribution at the time of lead placement [48]. During the generator placement, a second incision is made to create the IPG pocket. After tunneling the leads and connecting to the IPG, the wounds are irrigated and closed.

### 5.3. Postoperative Care

Patients are typically seen in the clinic a few days after the implantation to monitor wound integrity and to review the SCS program settings with the patient. The initial SCS settings commonly require adjustments in the first few weeks after implantation [55]. The electrodes implanted in the dorsal columns of the spinal cord deliver preprogrammed parameters to activate the spinal cord [56]. The SCS activates the Aβ sensory fibers in the dorsal columns, which produces analgesia through subsequent modulation of pain pathways in spinal gray matter [57]. Antidromic activation of Aβ fibers results in activation of the GABAergic interneurons of the dorsal column, inhibiting pain transmission. Orthodromic activation of Aβ sensory fibers modulates pain transmission through the descending serotonergic and noradrenergic pathways via the supraspinal feedback loop [27].

However, factors like breathing, heartbeat, and changing posture may make the delivery of therapy inconsistent by altering the distance between the spinal cord and the epidural SCS electrodes. Fixed output open-loop stimulation may result in under- or overstimulation as the spinal cord position fluctuates in the unchanged electric field [58]. When in closed-loop mode, the system can respond in real-time to correct variations in spinal cord activation, like body movement. ECAPs are a measure of the neural response to electrical stimulation from a given stimulation pulse. ECAPs may be used to confirm targeted fiber activation and confirm therapy delivery and adherence. The strength of each stimulus is adjusted automatically to maintain a consistent neural response [59].

SCS affects higher-order nociceptive processing at the segmental and cortical levels. Though segmental effects are difficult to determine in humans, effects on spinal reflexes can be inferred from neurophysiological tests. SCS has been found to inhibit sensorimotor reflexes like the Hoffman reflex in patients with lower limb pain [60]. The nociceptive sensorimotor reflex (RIII) is a polysynaptic spinal reflex that can also be considered as an objective measure of nociception and correlates positively with perceived pain. SCS has been shown to inhibit the RIII and stands as a promising test to establish optimal stimulation parameters [60]. Somatosensory excitation potentials (SSEPs) can be measured to predict pain relief [61]. However, SSEP suppression does not always correlate with clinical success. Imaging approaches like functional MRI, positron emission tomography, single photon emission computed tomography, and Xenon-133 inhalation have also been used to represent changes in cortical processing [60].

Programming adjustments may be necessary to optimize pain control over time [55]. During follow-up appointments, the severity of pain, sleep quality, and impact on daily function can be discussed. Patient-reported scoring systems like the BPI score, EQ-5D-5L score, ODI score, and PSQI score may be used to quantitatively measure quality of life improvements [4]. In addition, patients are monitored for common complications like infection, bleeding, hematoma formation, nerve damage, and lead migration [48]. Patients may require extra support like rehabilitation, physical therapy, and psychosocial support, and adequate follow-up should be ensured.

## 6. Clinical Data

The ability of the CL-SCS system to be considered as a technological successor to OL-SCS relies on robust clinical evidence that supports its efficacy and safety. Of note, the Initiative on Methods, Measurement, and Pain Assessment in Clinical Trials (IMMPACT) has released nearly annual consensus statements regarding the best practices for assessing patient-reported pain scores, for designing clinical trials to assess the efficacy of analgesic therapies, and for reviewing existing data from directed pain treatment and novel technologies [62]. Table 1 provides a brief summary of the two randomized controlled trials evaluating the CL-SCS system. Table 2 similarly summarizes several observational studies that evaluate CL-SCS in several pathophysiologic conditions. The studies included in these tables, as well as their clinical applicability, are elaborated in greater detail in this section.

### 6.1. Avalon Study

The Avalon study (ACTRN12615000713594) was one of the early clinical trials by Brooker et al. that assessed the efficacy and safety of the Evoke CL-SCS system. In 50 patients selected from 5 sites in Australia in accordance with IMMPACT guidelines, the Evoke SCS system was implanted, and the patients were followed for 24 months. Measurements of back and leg pain relief were recorded at baseline and at 3, 12, and 24 months after implantation and were based on patient-reported standardized pain metrics (i.e., BPI, ODI, PSQI, and EQ-5D-5L). The investigators found statistically significant and sustained reductions in pain scores from baseline levels, with 85% of patients exhibiting a response to treatment for an average of 77.3% reduction in pain scores. Secondary outcomes, such as quality of life, disability index, and sleep quality, were assessed as well and compared across these timepoints. Each of these holistic measures of pain was significantly improved from baseline at each time point, further suggesting a clinically significant and sustained effect. Additionally, Brooker et al. tracked changes in opioid use by the participants throughout the study. By the conclusion of the 24-month follow up, 82.8% of participants exhibited either a reduction or discontinuation of opioid use, with the average daily dose decreasing from 62.9 morphine milligram equivalents (MME) per day to 29.1 MME per day. Despite the single-armed nature of this study precluding comparison to traditional SCS technology, the consistent and robust responses across holistic measures of pain were promising results. Regarding safety, the study reported an adverse event rate similar to existing SCS devices, although follow-up length was limited to only 24 months [4].

### 6.2. Evoke Trial

Another primary study that assessed safety and efficacy endpoints was the Evoke trial (NCT02924129), which was a multicenter randomized controlled trial by Levy et al. initiated in 2019 to compare the efficacy and safety of CL-SCS to that of OL-SCS. Efficacy was assessed by comparing reductions in average patient-reported standardized pain metric scores (i.e., VAS, ODI, PSQI, and EQ-5D-5L) and the presence or absence of newly prescribed pharmacologic analgesics. The authors assessed safety by recording and comparing the number and severity of adverse events experienced by subjects in both the CL-SCS and OL-SCS treatment arms. These subjects, made up of 134 demographically similar individuals from 20 sites in the United States who experienced intractable trunk or limb pain, were selected in accordance with the IMMPACT guidelines and followed for up to 36 months. After a 1-week trial period during which responsiveness to SCS was assessed, they received an implanted Evoke-system SCS that was randomized to be programmed to a CL mode or an OL mode [72]. In as early as 12 months following initiation of the study, the CL group was noted to have spent significantly more time in the therapeutic window compared to the OL group, with far fewer rates of supratherapeutic stimulation amplitudes [16].

Upon conclusion of the Evoke study, there were clinically significant increases in the proportion of patients endorsing a ≥50% and a ≥80% improvement in back and limb pain in the CL group compared to the OL group, and there was no difference in the rate of adverse events between groups. Furthermore, while both groups experienced improvements in pain scores from baseline, patients in the CL group also exhibited superior improvements in secondary endpoints, including emotional function, sleep quality, and quality of life. Additionally, patients were permitted to crossover at the 24-month mark and were permitted to return to their initial treatment group after crossover. Patients who chose to crossover remained blinded; the primary reason given for choosing to crossover was curiosity and desire to experience an alternative therapy. Interestingly, of the patients who chose to crossover, 89% chose to complete the study in the CL setting despite remaining blinded throughout the process [63]. While this was a large randomized controlled trial that provided robust data, the limited follow-up period of 24 months and the demographic homogeneity of the sample limit clinical applicability to individual patients. Longer-term, more diverse randomized trials are needed to improve the clinical generalizability of the study’s findings.

### 6.3. ECAP Study

The ECAP study (NCT 04319887) was developed to identify the durability of an early response to trial periods of CL-SCS. Pope et al. identified 132 patients undergoing trials for SCS implantation and documented measures of SCS success (pain relief, functional improvement, and patient willingness to continue with SCS implantation) immediately after trial initiation (Day 0) and then at the end of the trial (EOT), approximately 1 week later. The investigators found a high positive predictive value, 98.4%, of a successful Day 0 evaluation predicting a successful EOT evaluation. Concurrently, the false positive rate was low, with only 5.6% of successful Day 0 evaluations “converting” to non-successful EOT evaluations [65]. This study demonstrated the fidelity of an early CL-SCS trial response to a successful trial and could support the transition to same-day trials in which immediate success after starting a trial CL-SCS system would be sufficient for completion of the trial and transition to permanent implantation. Because trial SCS systems consist of percutaneous epidural leads connected to wearable external stimulators, reducing the length of a trial period could reduce the time during which a patient is at risk of known complications (e.g., infection, bleeding) [73]. Of note, this finding is uniquely applicable to CL-SCS systems; in OL-SCS systems, immediate response to treatment may not accurately predict success outside of the clinic, where movements associated with activities of daily living (ADLs) that cannot be fully assessed in one clinic session may reduce the efficacy of the system and may not have similar consistency between Day 0 success measures and those at EOT.

### 6.4. ECHO-MAC Trial

The 2024 ECHO-MAC study (NCT04765735) also involved crossover between CL and OL settings in individuals with the Evoke-system and compared both subjective assessments of sensation as well as variability in measured ECAP amplitudes. By utilizing a 5-point Likert scale of overstimulation, study participants were asked to perform behaviors such as coughing, leg lifting, arm raising, torso twisting, and others common to ADLs and report intensity of symptoms of overstimulation (e.g., tingling, aching, shocking, etc.). The 42 study subjects were randomized to an OL-to-CL crossover group or a CL-to-OL crossover group. There was a 97.6% reduction in the number of overstimulation symptoms experienced during CL-mode compared to OL-mode. Consistency of spinal cord stimulation also varied significantly between CL and OL settings, with measured mean ECAP amplitudes of 24.5 μV (with SD 19.95 μV) during OL and 9.3 μV (with SD 8.72 μV) during CL and median stimulation amplitudes of 6.4 mA during OL and 3.96 mA during CL [64]. Patient preference also differed between groups to an extent strikingly similar to that demonstrated in the Evoke trial, with 88.1% of patients preferring CL compared to OL, and this was independent of crossover sequence. In this trial, there were no adverse events experienced in either study arm. However, the limited sample size of this randomized controlled trial should be noted and may decrease the generalizability of the findings.

### 6.5. Durability Study

The ongoing Durability study (NCT04627974) by Billet et al. is a prospective single-arm multicenter study in Europe assessing 70 patients with the CL-SCS implant. They primarily assess the change in pain scale from baseline, using patient-reported measures of pain severity, and additionally measure the rate of adverse events in a 60-month time frame [66]. This post-marketing assessment of the Evoke CL-SCS system will offer the longest-term assessment of its safety and the durability of its efficacy and is estimated to be completed in late 2027. Interim data analysis at 6 months has confirmed consistent device usage by patients, and an average improvement of 2.5 “minimal clinically important difference” points across the various holistic measures of pain severity [66]. While the improvement in follow-up length and the multicenter recruitment are strengths of this study, its single-arm design eliminates the potential to compare long-term efficacy and safety data of CL-SCS systems to OL-SCS systems.

### 6.6. Additional Studies

The prospective, single-center observational study of 22 patients with CL-SCS by Nijhuis et al. in 2024 corroborated the findings of the Evoke and Avalon trials, in which patients exhibited similar rates of satisfaction and average pain relief at both 3 and 12 months after implantation [67]. Additionally, the analysis by Levy et al. in 2024 compared a total of 180 patients from the Evoke trial and the ECAP and Durability studies at the common 3-month timepoint to determine the similarity in findings of efficacy and safety. The authors found no significant difference in maximal analgesic effect or neurophysiological ECAP amplitudes between these trials, suggesting adequate applicability of the Evoke randomized controlled trial to the “real world” Durability and ECAP studies. However, they uniquely noted that a neurophysiological ECAP dose ratio of 1.4, in which a stimulator dose that is 1.4 × the ECAP threshold, in mA, corresponded to the optimal improvement in analgesic efficacy [68].

The aforementioned clinical trials are predominantly limited to the application of CL-SCS in patients with intractable back or limb pain. Emerging data supporting the use of CL-SCS systems in distinct pain pathologies exist, such as pain caused by cancer, pelvic visceral pathology, or Raynaud’s phenomenon (RP). Chung et al. performed a retrospective analysis of four patients with cancer pain, comparing the analgesic properties of CL-SCS to dorsal root ganglion (DRG) stimulation. In this small sample, all patients expressed pain relief and endorsed functional improvement after both the DRG stimulation and CL-SCS, with three preferring the CL-SCS [69]. In a prospective study of 10 patients with RP, CL-SCS significantly improved the severity of exacerbations. However, the study was unable to demonstrate significant improvement in the frequency of exacerbations, demonstrating the need for future trials powered to evaluate these endpoints [70]. Chronic pelvic pain, which was previously described to be responsive to OL-SCS, has limited data associating its relief with CL-SCS [74]. Nonetheless, a case report was recently published that describes the complete alleviation of pain from CL-SCS implanted at T10-T11 in a patient with otherwise refractory pelvic pain [71]. These descriptions of novel applications of CL-SCS underscore the broad potential for its application in pain conditions beyond the well-studied back and limb pain. However, the broader applications described in the studies in this section have limited generalizability due to extremely small sample sizes. Additionally, because the studies listed in Table 2 are not randomized, the clinical superiority of CL-SCS in these groups cannot be adequately assessed. Larger randomized controlled trials are needed to develop clinically significant conclusions about the application of CL-SCS in different pathophysiologic states.

## 7. Discussion

As demonstrated by the existing data evaluating the safety and effectiveness of CL-SCS, it is currently a promising evolution of traditional OL-SCS systems and its widespread adoption into clinical practice is likely. However, there are several domains in which additional investigation is needed, and implementing CL-SCS systems requires overcoming some unique challenges. Specifically, a deeper understanding of how individual patient factors (e.g., age, comorbidities, medications) influence responses to CL-SCS systems would be important to better optimize therapy and predict outcomes in real-world applications [65]. Furthermore, much of the existing clinical data are limited to use in patients with lower back or lower extremity pain, with data examining CL-SCS used in other pain syndromes limited to small observational studies or case reports. Additional studies in patients with upper limb pain or visceral pain, with CL-SCS implantation in cervical or thoracic levels, are warranted. Interestingly, CL-SCS has been investigated in rat models of neuropathic pain phenomena, demonstrating marked analgesic capability in models with spared nerve injury, limiting the characteristic mechanical hypersensitivity and cold hyperalgesia [75]. This study lays the foundation for applying CL-SCS to humans suffering from neuropathic pain, although more data regarding its safety and clinical utility in humans would be needed before practical application.

Technological advancements can also be studied in CL-SCS systems, with retrograde ECAP measurements and sub-perception SCS augmentation. Retrograde ECAP values, which are measured distally to the stimulating electrode, have also been validated in rats as a way to provide supplemental feedback for a CL-SCS system [76]. Sub-perception SCS augmentation is described as low-frequency SCS (<200 Hz) that can provide rapid and effective analgesia by inducing surround-inhibition of dorsal horn neurons [77]. Because this stimulation occurred at frequencies below the perception threshold, the risk of generating paresthesia is theoretically minimized. While sub-perception SCS provides similar analgesia compared to conventional SCS in patients with refractory lower-back pain, as was demonstrated in a large randomized controlled by Sokal et al., no large randomized study has investigated whether sub-perception SCS significantly reduces paresthesia compared to conventional SCS [78]. Because CL-SCS systems have been shown to reduce the rate of supratherapeutic stimulation compared to OL-SCS, if sub-perception SCS is also shown to reduce paresthesia, the utilization of sub-perception SCS in CL systems may amplify this reduction.

Finally, CL-SCS systems have demonstrated cost-efficacy in their utilization to treat refractory lower back pain. In an analysis of patients approximately 5 years after either CL-SCS or OL-SCS implantation, CL-SCS was 92% more likely to be the cost-effective strategy, measured by cost per quality-adjusted life year [79]. A meta-analysis by Eldabe et al. corroborated this finding and also demonstrated superior cost-efficacy compared to conventional medical management, which was inferior to both CL and OL-SCS systems [80]. CL-SCS’s superior cost-efficacy, compared to both OL-SCS and conventional medical management, was consistent in both nonsurgical and post-surgical applications [79]. These findings, along with the broad potential for continued technological advancement, render CL-SCS systems a promising alternative to OL-SCS for procedural management of refractory lower-back pain. Nonetheless, the higher up-front cost of the CL-SCS systems, the need for training clinicians who are otherwise familiar with OL-SCS systems, and clinical data that are limited by sample size, demographic homogeneity, and short follow-up remain barriers to the widespread adoption of CL-SCS as a first-line management strategy.

## 8. Conclusions

CL-SCS represents a significant advancement in the management of chronic pain, particularly for lower back and limb pain, by dynamically adjusting stimulation parameters based on real-time neural feedback. Traditional open-loop SCS systems provide fixed-output stimulation, which fails to account for anatomical and physiological variability, leading to inconsistent pain relief. CL-SCS addresses these limitations by measuring and utilizing ECAPs to modulate stimulation amplitude, improving therapeutic efficacy while reducing overstimulation and discomfort. Clinical trials, such as the Avalon and Evoke studies, have demonstrated the superior pain relief, functional improvement, and reduced opioid utilization associated with CL-SCS compared to conventional SCS systems. Additionally, emerging research suggests potential applications in conditions beyond back pain, including neuropathic pain, cancer pain, and Raynaud’s phenomenon. While long-term data on safety and durability are still being gathered, early findings indicate that CL-SCS is more cost-effective than traditional SCS and conservative medical management, supporting its potential to improve the management of refractory chronic pain.

## Figures and Tables

**Table 1:** Randomized controlled trials evaluating the CL-SCS system.

Study	Publication Year	Study Type	Patients (*n*)	Endpoints	Findings
Evoke [63]	2024	Multicenter, Double-blinded, randomized controlled trial	134	Reduction of ≥ 50% in overall back and leg pain, objective neural activation amplitude	Significantly greater number of patients endorsing a ≥50% and a ≥80% improvement in back and limb pain with CL versus OL Significantly more time in the therapeutic window with CL versus OL Fewer supratherapeutic stimulation amplitudes in CL versus OL significant increases in the No difference in the rate of adverse events between groups
ECHO-MAC [64]	2024	Multicenter, single-blinded, crossover, randomized controlled trial	42	Overstimulation and understimulation during ADLs with CL versus OL, patient preference, ECAP amplitudes and dose consistency	97.6% reduction in overstimulation symptoms during CL-mode compared to OL-mode Mean ECAP amplitudes of 24.5 μV during OL and 9.3 μV during CL Median stimulation amplitudes of 6.4 mA during OL and 3.96 mA during CL 88.1% of patients preferred CL compared to OL

**Table 2:** Observational studies evaluating the CL-SCS system.

Study	Publication Year	Study Type	Patients (*n*)	Endpoints	Findings
Avalon [4]	2021	Prospective, multi-center, single-arm	50	Pain relief, opioid reduction	85% of patients exhibited a response to treatment 77.3% reduction in pain scores 82.8% of patients had either reduction or discontinuation of opioid use Decrease in average MME from 62.9 to 29.1 per day
ECAP [65]	2024	Prospective	132	PPV and FPR of a successful Day 0 trial evaluation predicting a successful EOT evaluation	98.4% PPV of a successful Day 0 evaluation predicting a successful EOT evaluation 5.6% FPR of successful Day 0 evaluations “converting” to non-successful EOT evaluations
Durability [66]	2025 (projected to finish in 2027)	Prospective, multi-center	70	Percent change in pain severity after implant	Interim 6 months analysis with confirmed consistent device usage by patients, and an average improvement of 2.5 MCID points across various holistic measures of pain severity
Nijhuis et al. [67]	2024	Prospective, multi-center	22	Difference in overall back/leg pain from baseline	Similar rates of satisfaction and average pain relief at both 3 and 12 months after implantation
Levy et al. [68]	2024	Post-hoc analysis	180	Changes in MAE, dose accuracy, and ratio during ECAP and Durability studies	No significant difference in MAE or neurophysiologic ECAP amplitudes between these trials (ECAP and Durability)
Chung et al. [69]	2025	Retrospective case series	4	Pain relief and functional improvement in DRG and CL-SCS	All patients expressed pain relief and endorsed functional improvement after both the DRG stimulation or CL-SCS, with 3 preferring the CL-SCS
Maciaczyk et al. [70]	2024	Prospective, single center	10	Severity and frequency of RP exacerbations after CL-SCS	CL-SCS significantly improved the severity of exacerbations. However, the study was unable to demonstrate significant improvement in frequency of exacerbations
Briggi et al. [71]	2024	Case report	1	N/A	Complete alleviation of pain from CL-SCS implanted at T10-T11 in a patient with otherwise refractory pelvic pain

## Data Availability

No new data were created or analyzed in this study.

## Abbreviations

The following abbreviations are used in this manuscript:

SCSs	Spinal cord stimulators
ECAP	Evoked compound action potential
CL-SCS	Closed-loop spinal cord stimulation
MeSHs	Medical Subject Headings
MRI	Magnetic resonance imaging
P1	Small positive spike
N1	Sharp negative spike
P2	Large positive spike
GA	General anesthesia
IPG	Implantable pulse generator
IMMPACTs	Initiative on Methods, Measurement, and Pain Assessment in Clinical Trials
MMEs	Morphine milligram equivalents
OL-SCS	Open-loop spinal cord stimulation
EOT	End of the trial
ADLs	Activities of daily living
RP	Raynaud’s phenomenon
DRG	Dorsal root ganglion
VAS	Visual Analog Scale
BPI	Brief pain inventory
EQ-5D-5L	EuroQOL instrument
ODI	Oswestry disability index
PSQI	Pittsburgh sleep quality index
SD	Standard deviation
GABA	Gamma-aminobutyric acid
RIII	Nociceptive sensorimotor reflex
SSEPs	Somatosensory excitation potentials
